# The incubus phenomenon: Prevalence, frequency and risk factors in psychiatric inpatients and university undergraduates

**DOI:** 10.3389/fpsyt.2022.1040769

**Published:** 2022-11-14

**Authors:** Marc L. Molendijk, Ouarda Bouachmir, Harriët Montagne, Laura Bouwman, Jan Dirk Blom

**Affiliations:** ^1^Department of Clinical Psychology, Institute of Psychology, Leiden University, Leiden, Netherlands; ^2^Leiden Institute for Brain and Cognition, Leiden University Medical Center, Leiden, Netherlands; ^3^Parnassia Psychiatric Institute, The Hague, Netherlands; ^4^Department of Psychiatry, University of Groningen, Groningen, Netherlands

**Keywords:** autoscopy, entity experience, false awakening, parasomnia, schizophrenia spectrum disorder, sensed presence, sleep paralysis, sleep-wake disorder

## Abstract

**Background:**

The incubus phenomenon is a paroxysmal sleep-related disorder characterized by the visuotactile sensation of a person or entity exerting pressure on one’s thorax during episodes of sleep paralysis and (apparent) wakefulness. This terrifying phenomenon is relatively unknown even though a previous meta-analysis indicated a lifetime prevalence of 0.11 for individuals in the general population and of 0.41 for selected at-risk groups, including people diagnosed with schizophrenia and students. Since the studies reviewed did not always make a strict distinction between the incubus phenomenon and isolated sleep paralysis, we carried out a cross-sectional study in a contemporary patient and student sample to attain current, more detailed data on the incubus phenomenon.

**Materials and methods:**

In a cross-sectional design, we used the *Waterloo Unusual Sleep Experience Questionnaire* (WUSEQ) to screen patients with severe psychiatric disorders and university undergraduates to establish and compare prevalence rates, frequencies of occurrence, and risk factors for the incubus phenomenon.

**Results:**

Having interviewed 749 people, comprising 606 students and 143 patients with a schizophrenia spectrum or related disorder who had been acutely admitted to a secluded nursing ward, we computed a reported lifetime prevalence of 0.12 and 0.09, respectively, which rates were not statistically different. In both groups, the phenomenon was more common in people with a non-Western European background. Risk factors noted for the students were the use of psychotropic medication and the lifetime presence of an anxiety disorder, eating disorder, or sleeping disorder. We found no associations with age or gender in either group.

**Conclusion:**

The 0.09 and 0.12 lifetime prevalence rates we recorded for the incubus phenomenon in students and psychiatric inpatients is substantially lower than the 0.41 found in an earlier meta-analysis. We tentatively attribute this difference to an overgeneralization in previous studies but also discuss alternative explanations. The elevated prevalence among non-Western European participants may well be due to the fact that the topic continues to be part of the cultural and religious heritage of many non-Western countries.

## Introduction

*Incubus* is Latin for night hag or nightmare. Stemming from the verb *incubare* (“to lie down upon”), the term once referred to a minor demon or fallen angel who, in the guise of a man, positioned itself on top of sleeping women to aggressively and/or sexually harass them. Others came in the guise of a woman who likewise beleaguered men. In the latter case, succubus used to be the preferred term (for an artistic impression of the succubus). The belief in incubi and succubi as living, metaphysical beings has its roots in ancient Greece and beyond (1, 2). Today, the phenomenon is conceptualized as a paroxysmal type of parasomnia. It is attributed to a dissociation of sleep phases, more specifically a state of (apparent) wakefulness accompanied by the intrusion of rapid-eye-movement (REM)-sleep-derived hallucinoid phenomena. It is believed that the aggressive content of these sensations originates from the threat-activated vigilance system (3–5).

Phenomenologically, the incubus phenomenon is characterized by a sensation of pressure on the chest that is accompanied by sleep paralysis and compound hallucinoid experiences involving a creature positioning itself on the thorax, exerting pressure, and carrying out aggressive and/or sexual acts. In the majority of reported cases, the sense of dread tends to be extreme and is probably heightened by the inability to move or make any sounds. Occasionally, people report having been able to move their eyes, but, due to the physiological atonia of striate muscles during REM sleep, they are usually unable to make any other movements. Symptoms may additionally include a sensed presence, auditory hallucinations, tactile hallucinations, sexual hallucinations, olfactory hallucinations, elevator feelings, out-of-body experiences, and vegetative symptoms such as tachycardia, hypertension, piloerection, a cold sweat, and the sensation of suffocation (3). Attacks tend to last seconds to minutes and to end abruptly, leaving the “victim” behind in a state of terror and exhaustion. Risk factors include narcolepsy and other sleep disorders (6, 7), irregular sleep (8), a supine sleeping position [with or without airway obstruction and apnoea (9)], post-traumatic stress disorder (PTSD) (10–12), psychosis (13), anxiety disorders (14), exploding head syndrome (15), a history of sexual abuse (16, 17), stress (6), physical illness (6), and intoxication with alcohol (18) or amphetamines (19).

### Rationale

Although the incubus phenomenon has been described since antiquity, it took considerable time before it gained scientific interest (20). A recent search in PubMed yielded 25 results, with the oldest publication dating back to 1995. By contrast, the search term “sleep paralysis” yielded 1,311 results, with the oldest study having been published in 1933. The incubus phenomenon was never included in major classifications such as the *International Classification of Diseases* [ICD-11 (21)] or the *Diagnostic and Statistical Manual of Mental Disorders* [DSM-5-TR (22)]. Perhaps due to this lack of scientific attention–and the ensuing lack of familiarity among clinicians and researchers–the two phenomena appear to have been treated as identical or at least interchangeable in a number of older studies. This may explain the relatively wide range of prevalence figures reported in previous research. Another explanation for between-study heterogeneity in outcomes could be regional or cultural differences regarding the existence of the phenomenon or the meaning attached to it. Taking these older studies as the basis for a systematic review and meta-analysis, our group (23) found the reported lifetime prevalence of the incubus phenomenon to be 0.11 in the general population and 0.41 for at-risk groups (i.e., individuals diagnosed with narcolepsy or schizophrenia spectrum disorder, refugees, and students). Although the studies that were included appeared to have made a careful distinction between the incubus phenomenon and isolated sleep paralysis, the question remained whether the outcome was fully reliable.

### Objective

By using a questionnaire that allows for a proper distinction between isolated sleep paralysis and the incubus phenomenon, in the present study we aimed to establish the lifetime prevalence of the incubus phenomenon in two specific populations: patients diagnosed with severe psychiatric disorders and undergraduate university students.

## Materials and methods

In a cross-sectional design we investigated the prevalence and the frequency of occurrence of the incubus phenomenon in a consecutive group of acutely ill patients diagnosed with a schizophrenia spectrum disorder and related psychiatric disorders, and a group of university undergraduates. Since the applied measures and procedures differed somewhat for the two groups, they will be described separately.

### Participants, instruments, and procedure

#### Inpatient population

From 2019 through to 2021 we screened all patients newly admitted to the High Care Department of Parnassia Psychiatric Institute for sleep paralysis and the incubus phenomenon in The Hague, Netherlands. The High Care Department is a secluded nursing ward for adults diagnosed primarily with psychotic disorders, some 50% of whom are sectioned under the Dutch Mental Health Act.

We used the *Waterloo Unusual Sleep Experience Questionnaire* (WUSEQ) (24) to assess the patients as to their lifetime experiences of these phenomena as well as their vividness and intensity, the latter of which rated on a 7-point Likert scale. During the semi-structured interview various types of hypnagogic and hypnopompic hallucinoid experiences were addressed (including sensed presence) and the emotional reactions to them (e.g., fear, panic, and anger). The WUSEQ was administered by two trained nurses (OB and LB) and a psychiatric resident (HM), all of whom had ample experience in the management and treatment of this complex patient population. Whenever they were in doubt, they discussed the results with an experienced clinical psychiatrist (JDB). Participants were further asked about their age, gender, drug use, and their and their parents’ country of birth. Details on medication use and diagnoses were derived from their medical records.

The study received ethical approval from the Ethical Review Board of Leiden University Medical Centre, Netherlands (number: NL66211.058.18). Participation was voluntary and all participating patients provided signed informed consent.

#### Student population

From 2017 through to 2020, we screened four subsequent groups of undergraduate psychology students at Leiden University who attended a lecture on psychopathology presented by the first author (MM).

Because of time constraints, the students completed an abridged version of the WUSEQ that exclusively addressed the items gauging sleep paralysis, sensed presence, and the incubus phenomenon, and questions pertaining to the vivacity of the phenomena and the ensuing distress. We had added open questions about the students’ preferred sleeping positions (i.e., on their stomach, their back, their left or right side, or variable), the estimated duration of the incubus experience(s), and any emotions evoked. Finally, the students were also asked to provide their age, gender, country of birth, the country of birth of their parents, past or current psychiatric history, and past or current drug and medication use.

#### Ethical approval

This second part of the study was approved by the Ethical Review Board of the Department of Psychology of Leiden University (number: 2017-09-13-M.L. Molendijk-V1-806). Participation was voluntary and all students gave their signed informed consent prior to their participation.

#### Statistical analyses

For our analyses we used *Stata Statistical Software: Release 17* (25). In what follows, we report on lifetime prevalences (with 95% confidence intervals) of the incubus phenomenon for all participants. Associations with demographic and clinical variables were examined by means of cross-tabulation for categorical variables and independent *t*-tests for continuous variables. Statistical significance was set at *P* < 0.05.

## Results

### Inpatient population

In total, 143 adult psychiatric patients with an average age of 47 years (SD = 14.0) completed the WUSEQ-guided interview, of whom 47% were female, while 43% had a Western European background. Of the 45% participants with a different (parental) background 14% (originally) hailed from Surinam, 11% from Turkey, and 4% from Morocco. Information on the country of origin was missing for 12% of the participants. The primary diagnosis for the greater majority was a schizophrenia spectrum disorder (69%), followed by bipolar disorder (7%), and major depressive disorder (3%), with 2% reporting to have been diagnosed with a sleeping disorder. Diagnostic information was missing for 10% of the participants. Of all clinical respondents, 24% reported to have used illicit substances, most often cannabis (17%). There was no data available on drug use for 19% of the participants. All patients interviewed used psychotropic medication.

#### Sleep paralysis and the incubus phenomenon

Of the 143 patients interviewed 12% reported to have experienced sleep paralysis at least once during their lives. All of these positive responders also reported to have experienced an incubus phenomenon, 3% only once, another 3% a few times, and 6% multiple times on a monthly or weekly basis. The average age at which the incubus phenomenon had first been experienced was 27 years (SD = 16, range 2–52). We found no associations between the incubus phenomenon and age, gender, position when falling asleep, diagnosis, and medication use (Table 1). Experiences of the incubus phenomenon were less frequent among the respondents of Western European descent (5%) relative to the rate reported by patients from other origins (16%) (*P*-value for the difference = 0.006). The participants’ backgrounds were too diverse to perform any further analyses.

**TABLE 1:** The incubus phenomenon and associated factors*.

	Patient population (*N* = 143)		Student population (*N* = 606)	
	No incubus (*n* = 126)	Incubus (*n* = 17)	No incubus (*n* = 535)	Incubus (*n* = 71)
Age	47.9 (14.2)	41.5 (11.9)	22.2 (4.3)	21.6 (2.3)
Female	55 (44%)	12 (71%)	459 (86%)	61 (86%)
W-European	59 (54%)	3 (19%)***	398 (74%)	50 (70%)
Medication use	Basically all	Basically all	17 (3%)	5 (7%)
Psychiatric disorder	126 (100%)	17 (100%)	91 (17%)	21 (30%)**
Sleeping disorder	55 (44%)	12 (71%)	8 (2%)	3 (4%)
Drug use	3 (2%)	0 (0%)	58 (11%)	10 (14%)

*Categorical variables are presented as *n* and percentages of the column.

**P < 0.01; ***P < 0.001.

Of the positive respondents, 71% denoted the incubus phenomenon as having been lively or very lively and 53% tried to talk or scream during the attack, only to find that they were unable to do so. In the same group, 65% reported the additional experience of sensing the presence of a person or entity. All respondents rated the above experiences as negative, with the majority reporting having had the feeling of being choked and/or dying. Table 2 lists the experiences recounted.

**TABLE 2:** Verbatim emotional reactions to the incubus phenomenon in the patient group (*n* = 17).

During the experience:	Never	Sometimes	Always	Don’t know
*I had the experience that I choked*	4 (24%)	5 (29%)	8 (47%)	0 (0%)
*I heard strange noises*	11 (65%)	3 (18%)	2 (12%)	1 (6%)
*I felt like I was going to die*	7 (41%)	4 (24%)	6 (35%)	0 (0%)
*My body felt numb*	7 (41%)	4 (24%)	5 (29%)	1 (6%)
*It was as if I was out of my body*	11 (65%)	6 (35%)	2 (12%)	0 (0%)
*It was as if I could look at myself*	13 (76%)	4 (24%)	0 (0%)	0 (0%)
*I smelt a strange odour*	14 (82%)	1 (6%)	2 (12%)	0 (0%)
*It felt as if I were moving up and down*	14 (82%)	2 (12%)	1 (6%)	0 (0%)
*It felt as if sheets/blankets were moving*	13 (77%)	3 (18%)	1 (6%)	0 (0%)
*I had the feeling that I was falling*	9 (53%)	7 (41%)	1 (6%)	0 (0%)
*I had the idea to have moved, only to later notice that this was not the case*	12 (71%)	2 (12%)	2 (12%)	1 (6%)
*I had the feeling that I was being strangled or that I choked*	8 (47%)	5 (29%)	4 (24%)	1 (6%)
*I had the feeling that I was flying*	16 (94%)	1 (6%)	0 (0%)	0 (0%)
*I had the feeling that I was turning around*	14 (82%)	3 (18%)	0 (0%)	0 (0%)
*It was as if someone/something touched me*	4 (24%)	4 (24%)	9 (53%)	0 (0%)
*I could open my eyes*	4 (24%)	6 (35%)	7 (41%)	0 (0%)
*I had feelings of happiness/delight/bliss*	15 (88%)	2 (12%)	0 (0%)	0 (0%)
*I experienced pain*	5 (29%)	6 (35%)	6 (35%)	0 (0%)
*I felt cold*	8 (47%)	6 (35%)	3 (18%)	0 (0%)
*I felt sad*	5 (29%)	4 (24%)	8 (47%)	0 (0%)
*I felt anger*	6 (35%)	7 (41%)	4 (24%)	0 (0%)
*I had erotic/sexual feelings*	13 (77%)	4 (24%)	0 (0%)	0 (0%)
*It was as if I were trembling/shivering*	7 (77%)	5 (29%)	4 (24%)	1 (6%)

### Student population

Of the 606 students having completed the questionnaire, 86% were female. The students’ average age was 22 years (SD = 4) and the large majority (74%) had a Western European background. Nineteen percent reported having been diagnosed with a current or past psychiatric disorder. The most frequently self-reported disorders were depressive disorder (9%), anxiety disorder (5%), attention-deficit hyperactivity disorder (ADHD 3%), and eating disorder (3%). Notably, 6% reported a sleeping disorder, particularly insomnia. There was no data available on drug use for 19% of the participants. Four percent reported the use of psychotropic medication and 11% the use of illicit substances, most frequently cannabis (8%).

#### Sleep paralysis and the incubus phenomenon

In the student group, 25% reported to have experienced sleep paralysis, with 17% recounting a single event, 7% several, and 2% multiple events on a monthly or weekly basis. Sleep paralysis was reported more often by male respondents and by respondents (originally coming) from Africa, Asia, and South-America. The same held true for self-reported PTSD, anxiety disorder, and sleeping disorder.

Symptoms of the incubus phenomenon were reported by 9%, where 3% had experienced these only once, another 3% several times, and yet another 3% more frequently, on a monthly or even weekly basis. On average, these experiences were characterized as rather lively and somewhat distressing. The duration of the episodes ranged from an estimated 10 s to 15 min (average 4.22 min, SD = 3.31). Participants reporting the use of psychotropic medication and those mentioning an anxiety disorder, eating disorder, and sleep disorder were more likely to have experienced the phenomenon. We did not investigate reasons underlying these differences, but suspect that they could have emerged due to either stress (6) or a poor sleep hygiene (8). The incubus phenomenon was relatively uncommon among students of Western European descent and more common among those with a South-American, Caribbean, and Asian background. Age, gender, and posture when falling asleep were unrelated to the reported experiences with the incubus phenomenon.

## Discussion

### Summary of main findings

Even though, on average, the students we screened in this study were significantly younger than the patients (22 vs. 47 years of age), we found the lifetime prevalence of sleep paralysis to be higher in this relatively healthy group (*n* = 606) than it was in the 143 participants with severe psychiatric disorders (0.25 vs. 0.12). Prevalence rates for the incubus phenomenon, however, did not differ significantly between the two groups (0.09 and 0.12, respectively). All patients reporting sleep paralysis also reported on one or more episodes involving an incubus phenomenon, whereas in the student group this was only 36%. The self-rated severity and impact of the episode(s) were both higher in the patient group, as was the proportion of recurring monthly or weekly events (6 vs. 3% in the student group). In both groups the incubus phenomenon was reported more often by respondents with a non-Western European background. This was also the case for students mentioning a past or current psychiatric disorder (11%) and those (additionally) reporting taking psychotropic medication.

### Stochastic process

Since all participants having experienced an incubus phenomenon also reported sleep paralysis, it seems safe to conclude that sleep paralysis is a necessary condition for the incubus phenomenon to manifest itself. This is in line with previous research, including the lack of case reports on incubus phenomena in the absence of sleep paralysis. The incubus phenomenon can thus be characterized as an entity experience superimposed on a state of sleep paralysis. Like other entity experiences, such as lilliputian hallucinations (26), dimethyltryptamine-induced machine elves (27), God experiences (28), and hallucinations attributed to *jinn* (29), there appears to be a fixed order to the sensory modalities that are being recruited, at least during the initial stages of the process, which invariably starts out with the somatosensory modality (muscle atonia), followed by the tactile modality (the sense of pressure being exerted on the chest), and then the visual modality (seeing a person or entity sitting on top of one’s body). During the stages that follow, modalities such as the sexual, auditory, olfactory, and vestibular modalities may or may not be recruited. Whether there is a fixed pattern to these latter stages is in need of further study, but, in conformity with the nature of stochastic processes, they do not seem to be activated without the prior recruitment of the somatosensory, tactile, and visual modalities.

### Frequency

A novel finding of our study is that in a third of the students and half of the patients reporting the incubus phenomenon, experiences occurred monthly or even weekly. These are exceptionally high rates, especially in the context of earlier findings that most instances of the incubus phenomenon are non-recurring [e.g., (30)]. Since all respondents in the clinical group moreover rated their experiences as negative, there appears to be no habituation or spontaneous response extinction over time, while Mayer and Fuhrmann (31) propose that an increased frequency of sleep paralysis (not necessarily complicated by an incubus phenomenon) leads to “habituation and de-dramatization” in at least some of the people affected. Since negative attributions have been found to be predictors of schizophrenia spectrum disorders, especially in the context of voice hearing (32)–where the risk of transition to psychosis over time tends to be smaller for people with positive thoughts about their auditory hallucinations than it is in those harboring negative thoughts–these traits may also play an important role in the mediation of incubus phenomena.

### Historical and cultural aspects

Historically, in Western countries the incubus phenomenon has had many names, among which are incubus, incubus experience, *incubo*, familiar, night hag, (classical) nightmare, *pnigalion*, and *pnigalium* (33). Sharpless and Doghramji (2) identified 117 different names in as many languages, confirming that the phenomenon is widely–and perhaps even universally–known. The reason why the non-Western European participants in our study reported the incubus phenomenon more often may well be due to the fact that the topic continues to be part of the cultural and religious heritage of many non-Western countries, whereas in Western Europe the belief in actual incubi and succubi gradually faded during the Enlightenment and virtually disappeared in the 19th Century (34). Taking into account that in Western countries the phenomenon did not resurface until the 1990s, and then only as a neuroscientific concept and thus remaining little known to the general population, it is obvious that it plays no role of significance in Western popular thinking. This then explains why almost none of the Western European participants to our study had a name for their experiences with the phenomenon, whereas many of the non-Western Europeans had. Muslim respondents of Turkish descent, for example, often spontaneously offered the term *karabasan*, while participants of Chinese descent readily talked of *bei guai chaak* or *bèi guĭ yā*. That said, not all of the participants were able to specify what it was that seemed to have harassed them. One participant appeared to describe a variant of autoscopy when she asserted, “*It is not a thing or being; the pressure on my chest is there because I myself sit on my chest*” [*sic*]. Another participant appeared to describe an extracampine hallucination when she stated, “*I get vibrations around my neck and ears that are experienced as screams*” [*sic*].

### Neuropsychology

If it is true that familiarity with the incubus theme may increase the chance of actually experiencing the phenomenon, this suggests a mediating role for catastrophic thoughts that arise during (physiological) phases of sleep paralysis, in the sense that people becoming aware of a normal state of sleep atonia may recognize this as a prelude to a full-blown incubus attack and subsequently start to panic, thereby paradoxically promoting its further development. This seems to be confirmed by at least one of our participants, who said, “*When I get nervous during the paralysed feeling I feel the pressure [on the chest]. When I think about something relaxing, it all goes away*” [*sic*]. This coincides with the role suggested for a hypervigilant state initiated in the midbrain (35), with psychological as well as neurobiological components. Although this is in need of further study, this might also explain why all the participants in the patient group who experienced a sleep paralysis went on to develop an incubus phenomenon (vs. 36% of those in the student group): being acutely and severely mentally ill, their overall stress levels may have been higher, and therefore also their propensity to generate catastrophic thoughts.

### Neurobiology

The underlying theory of the incubus phenomenon, formulated by Cheyne et al. (35), has become known as the experiential source hypothesis, which holds that the neurobiological underpinnings of the phenomenon are hard-wired, whereas its phenomenological features depend on attributional styles, which are in turn steeped in cultural and religious values. The threat-activated vigilance system is a neural network in which the midbrain and the amygdala play important roles. From an evolutionary perspective, the system is pivotal to the fight-or-flight response, designed to increase our chances of survival in the face of imminent danger. In that light, the incubus phenomenon can be interpreted as a false-positive response to a perceived threat that is not actually there, which traps the sleeping individual in a series of self-reinforcing arousal mechanisms.

### Waking or dreaming?

Whether people are asleep or awake during an incubus attack has long been a subject of debate. Spectral EEG analyses indicate that sleep paralysis has the same EEG power spectrum as false awakenings when people are convinced that they are up and about (e.g., walking to the bathroom, shaving, preparing breakfast) while they are actually still asleep and lying in their beds (36). The answer to whether people experiencing an incubus phenomenon are actually awake or asleep may then perhaps be found in this finding in that they are still asleep even though they experience their state as one of wakefulness. This would also explain why some people report to “wake up in a different place” while experiencing an incubus phenomenon. As one of our Moroccan participants asserted, a *jinn* would only mount his chest when he inexplicably woke up in an ice cave. This is in line with the current conception of sleep paralysis as a type of parasomnia, i.e., and intermediary state between a proper wakefulness and proper REM sleep, with intermingled features of both consciousness states in which sleep itself is envisaged as a local rather than a global cerebral phenomenon.

### Recommendations for further research and clinical practice

On the basis of our findings, we recommend further research on the prevalence, frequency, severity, and associated levels of distress of the incubus phenomenon in populations traditionally considered at higher risk, such as refugees and people diagnosed with narcolepsy. Given that the student and patient groups that we studied had an almost four times lower risk than historically reported but also a higher frequency of severe and frequently recurring attacks, it would be highly informative to establish whether this also applies to other groups, including those with sleep disorders in the broadest sense (i.e., not just insomnia). As a corollary, we recommend to improve the dissemination of knowledge regarding the incubus phenomenon, especially in the light of our hypothesis that catastrophic thoughts may promote the transition from sleep paralysis to full-blown, sometimes highly distressing attacks. Both health professionals and the general public may benefit from a greater awareness of and more insight into the phenomenon and its proposed working mechanisms. It would be of great help if neurological, psychiatric, and psychological textbooks were to include the phenomenon and if future editions of major diagnostic classifications such as the ICD and DSM would document it as an entity experience superimposed on episodes of sleep paralysis and include sound diagnostic criteria for a proper distinction between clinical and non-clinical cases. Clinically, there is still much work to do before we will be able to formulate evidence-based guidelines recommending whether treatment should involve both pharmacotherapy and psychological counseling, with programmes aimed at reducing or eliminating risk factors.

### Limitations

Our study has several limitations. First, the two groups under study differed significantly regarding mean age and gender distribution. As a consequence, our between-group comparison of prevalence rates was not fully satisfactory. What complicates this further, is that the data we derived from the student population might hardly be taken as general-population findings. After all, the majority of the respondents in this group were women–who might or might not be more prone to self-focusing on their health issues–and all of them were students, which implies that there IQ was above average. A second limitation is that we have no data on the nature and severity of the psychopathology in the 19% of students diagnosed with a current or past psychiatric disorder, which might have allowed us to create an intermediate group on the continuum between healthy participants and institutionalized psychiatric patients. A third limitation is the (modest number of) missing data in each group. Fourthly, what also stood in the way of a proper comparison is that the assessment methods (e.g., interview vs. questionnaire) differed somewhat for the two study populations. Fifthly, cases in the clinical group may have been missed as a result of underreporting. It is not uncommon for patients in acute psychotic or affective states to be so absorbed by other symptoms (e.g., voices, ruminations) that they are either incapable of shifting their focus of attention to the topic at hand or fail to register or fully comprehend the questions being asked. When we had the impression that either situation had played a role, we made sure to interview the patients concerned again at a later stage during their convalescence. Still, we cannot rule out that the relatively low incident rates for sleep paralysis and incubus experiences in this group are partly attributable to patients’ preoccupations with other pathology.

## Conclusion

Contrary to previous findings, our cross-sectional study of 606 university undergraduates and 143 psychiatric inpatients indicates that the lifetime prevalence of the incubus phenomenon is (i) equally high for (relatively) healthy students and patients with severe psychiatric disorders recently admitted to a secluded nursing ward, and (ii) almost four times lower than previously reported for either group. Possible explanations for these deviating findings are (i) underreporting by the patients in our study due to a preoccupation with current (more) severe psychopathological phenomena, and (ii) poor differentiation between the incubus phenomenon and isolated sleep paralysis in previous studies.

## Data availability statement

The raw data supporting the conclusions of this article will be made available by the authors, without undue reservation.

## Ethics statement

The studies involving human participants were reviewed and approved by Ethical Review Board of Leiden University Medical Centre, Netherlands (number: NL66211.058.18); Ethical Review Board of the Department of Psychology of Leiden University (number: 2017-09-13-M.L. Molendijk-V1-806). The patients/participants provided their written informed consent to participate in this study.

## Author contributions

MM contributed to the conception and design of the work, and to the acquisition, analysis, and interpretation of data for the work, drafted and revised the work, gave final approval for the final version to be published, and agreed to be accountable for all aspects of the work in ensuring that questions related to the accuracy or integrity of any part of the work are appropriately investigated and resolved. OB, HM, and LB contributed to the acquisition and interpretation of data for the work, revised the work, gave final approval for the final version to be published, and agreed to be accountable for all aspects of the work in ensuring that questions related to the accuracy or integrity of any part of the work are appropriately investigated and resolved. JDB contributed to the conception and design of the work, and to the analysis and interpretation of data for the work, drafted and revised the work, gave final approval for the final version to be published, and agreed to be accountable for all aspects of the work in ensuring that questions related to the accuracy or integrity of any part of the work are appropriately investigated and resolved. All authors contributed to the article and approved the submitted version.
